# Molecular Recognition: Perspective and a New Approach

**DOI:** 10.3390/s21082757

**Published:** 2021-04-14

**Authors:** W. Rudolf Seitz, Casey J. Grenier, John R. Csoros, Rongfang Yang, Tianyu Ren

**Affiliations:** 1 Department of Chemistry, University of New Hampshire, Durham, NH 03824, USA; jrz56@wildcats.unh.edu (J.R.C.); tr1063@wildcats.unh.edu (T.R.); 2 Tekscan, Inc., 307 W. First St., Boston, MA 02127, USA; cgren1@gmail.com; 3 Community College of Rhode Island, 400 East Ave., Warwick, RI 02886, USA; ryang@CCRI.edu

**Keywords:** molecular recognition, templating, molecularly imprinted polymers, chemical sensors

## Abstract

This perspective presents an overview of approaches to the preparation of molecular recognition agents for chemical sensing. These approaches include chemical synthesis, using catalysts from biological systems, partitioning, aptamers, antibodies and molecularly imprinted polymers. The latter three approaches are general in that they can be applied with a large number of analytes, both proteins and smaller molecules like drugs and hormones. Aptamers and antibodies bind analytes rapidly while molecularly imprinted polymers bind much more slowly. Most molecularly imprinted polymers, formed by polymerizing in the presence of a template, contain a high level of covalent crosslinker that causes the polymer to form a separate phase. This results in a material that is rigid with low affinity for analyte and slow binding kinetics. Our approach to templating is to use predominantly or exclusively noncovalent crosslinks. This results in soluble templated polymers that bind analyte rapidly with high affinity. The biggest challenge of this approach is that the chains are tangled when the templated polymer is dissolved in water, blocking access to binding sites.

## 1. Introduction

This paper presents an overview of the preparation and performance of molecular recognition elements that are used in chemical sensors. We are particularly interested in approaches that are general, i.e., can be applied to a wide number of possible analytes. We use this overview to provide a context for our recent work on templated polymers with predominantly or exclusively noncovalent crosslinks.

This perspective will consider the importance of molecular recognition for chemical sensing. It will review different ways in which the molecular recognition element can interact with an analyte. It will distinguish two approaches that can be applied to a large number of analytes. One of these, that we call the “shotgun” approach, involves a library of 10^12^ or more molecular recognition elements. Those that bind to the analyte are amplified and recovered by a separation method. The other involves polymerizing around a template. Usually these polymers include a high degree of covalent crosslinking so that the template binding site holds it conformation. Our research goal has been to explore the potential of using noncovalent crosslinks in place of covalent crosslinks to hold binding conformation. Our preliminary results suggest that at the more flexible site the results from this approach bind with high affinity at much faster rates than polymers with a high degree of covalent crosslinking [1].

## 2. Molecular Recognition in Chemical Sensing

Chemical Sensors are often considered to consist of two elements, a recognition element and a transducer. The function of the recognition element is to interact with the analyte and to undergo some sort of change that can be detected electrically or optically. This is converted to a measurable signal by the transducer.

A biosensor is a chemical sensor that uses a molecular recognition element that is derived from a biological system. Most chemical sensors that respond to aqueous solutes are biosensors. The IUPAC definition of a biosensor is a device that uses a biological recognition element with a transducer [2].

The recognition element can interact with the analyte to be sensed in several ways. In ion selective electrodes, the recognition element is often a lipophilic ionophore that can interact with ions and selectively transport them across a membrane [3,4]. The measured parameter is the potential across the membrane. This is a steady state rather than an equilibrium measurement. However, the time scale of the measurement is so much shorter than the time scale of ion transport that the concentrations on either side of the membrane change only slightly. This becomes an important issue only when trying to use an ion selective electrode to measure very low analyte concentrations.

The recognition element can be an enzyme that selectively catalyzes a reaction involving the analyte [5,6]. This approach has the attractive feature that transduction can be based on measuring either the amount of product formed or the amount of a substrate consumed by the enzyme catalyzed reaction. An example of this is the glucose electrode based on the immobilized enzyme, glucose oxidase. This enzyme catalyzes the reaction between glucose and oxygen to form gluconic acid and hydrogen peroxide. The amount of oxygen consumed or hydrogen peroxide produced can be measured by voltammetry. This type of electrode involves a steady state rather than an equilibrium. While there are several successful examples of this approach, it is limited by the availability of enzymes.

The recognition element can simply be a phase [7]. The response is based on analyte partitioning between the phase and the sample. Selective analysis is achieved by using an array of sensors with different phases. Mathematical analysis exploits small differences in the tendency of different recognition elements to interact with different analytes. This approach is employed in what are called “electronic noses”, sensor arrays that work well when applied to gas phase samples.

In this perspective, we are more interested in molecular recognition elements that are formed by approaches that can be applied to a large number of analytes. These include antibodies, i.e., proteins synthesized by the immune system in response to foreign substances, aptamers, i.e., single strand DNA molecules that adopt unique conformations depending on the nucleic acid sequence, and templated polymers. The interactions of these molecular recognition elements with the analyte involves a chemical equilibrium that can be characterized by an equilibrium constant.

In practice, most chemical sensors that respond continuously to analyte concentration are based on steady state measurements, e.g., ion selective electrodes, or catalysis, e.g., enzyme electrodes. However, low cost devices that measure analyte concentrations on a one-time basis may also be considered chemical sensors if they can be applied on-site with measured values that can be acquired quickly enough to follow and adjust a chemical system.

There is no reason why molecular recognition elements that interact with an analyte via a chemical equilibrium cannot be used for continuous sensing. However, this requires the appropriate equilibrium constant for binding and binding kinetics that are fast enough so that the sensor responds quickly enough to be practical.

## 3. Figures of Merit for Equilibrium Binding by Molecular Recognition Elements

There are several figures of merit for molecular recognition elements that interact with the analyte via a chemical equilibrium. These include binding affinity, binding kinetics, selectivity, stability and the magnitude of the electrical or optical change that accompanies binding. In this perspective, we will concentrate on binding affinity and kinetics. These are general characteristics that can be used to compare different approaches. Stability is a function of the particular recognition element. The degree of selectivity required for a particular application depends upon the composition of a sample.

The magnitude of the electrical or optical changes that accompany binding also depends on the particular molecular recognition element. However, we believe that the research approach that we are pursuing, as described in Section 7 of this perspective, offers the possibility of larger electrical or optical changes than other molecular recognition elements.

The dominant molecular recognition elements for on-site chemical analysis are antibodies, proteins synthesized by the immune system in response to foreign invaders. These have been successfully applied to analysis because they bind with high affinity and selectivity. When an antibody binds its target, generally known as an antigen, this leads to a change in size and refractive index, which can serve as the basis for detection. However, the reaction between an antibody and antigen is usually visualized indirectly, e.g., by measuring antigen concentration based on its ability to displace a labelled antigen from an antibody.

- Equilibrium Constant for the Binding Reaction

The interaction between analyte, A, and Molecular recognition element, MR, may be described:

A + MR ←→ AMR

where A stands for Analyte and MR stands for Molecular Recognition agent. The equilibrium constant for this reaction is an affinity constant.

K_affinity_ = [AMR]/[A][MR] = 1/K_D_

It will have units of M^−1^. This equilibrium is more often characterized by a dissociation constant, K_D_, which is the reciprocal of the affinity constant and has units of concentration.

The importance of the dissociation constant for chemical sensing is best appreciated by rearranging the binding constant expression to the following:

[AMR]/[MR] = K_affinity_[A] = [A]/K_D_

The ratio of molecular recognition element bound to analyte to free molecular recognition element depends on the analyte concentration and the dissociation constant. If the dissociation constant is much larger than the analyte concentration, then very little analyte is bound to the molecular recognition agent. If this is the case, sensing fails unless it is possible to detect a small percentage of binding. For example, if K_d_ is 1 micromolar and the analyte concentration is 10 nanomolar (10 × 10^−9^ M), then only approximately 1% of the molecular recognition agent will be bound to analyte at equilibrium.

On the other hand if K_D_ is much less than the analyte concentration, then the molecular recognition element will be essentially completely bound. This situation does make it possible to use the molecular recognition for one-time analysis but is not useful for continuous sensing. Continuous requires that K_D_ and the analyte concentration be similar in magnitude.

B. Binding Kinetics

It is important to remember that the affinity constant is equal to the ratio of the rate constant for the forward reaction, binding of the analyte by the molecular recognition element, to the rate constant for the back reaction, dissociation of the analyte from the molecular recognition element.

The forward rate establishes the time required for a one-time measurement. The backward rate establishes whether a particular molecular recognition element will be able to follow changes in analyte concentration at a practical rate for continuous sensing. Another way of saying this is that the backward or dissociation rate establishes the response time if a molecular recognition element is to be used for continuous sensing.

It follows from the equilibrium equations above that the lower the analyte concentration, the smaller the dissociation constant that is required for continuous sensing. This in turn requires a slower back reaction since the forward reaction rate is limited by the number of collisions between the molecular recognition element and the analyte with the appropriate orientation for binding. This means that the lower the analyte concentration, the slower the response will be.

## 4. Strategies for Preparing Molecular Recognition Elements

- Chemical Synthesis

If you pick a target analyte, it is possible to synthesize a molecular recognition element that selectively binds this analyte. Chemical synthesis has successfully prepared a variety of lipophilic ionophores that selectively transport ions across membranes [2]. These have been used as the molecular recognition agents in ion selective electrodes.

However, chemical synthesis is not easily applied to most analytes and is not a practical approach for preparing most molecular recognition agents.

B. Drawing from Nature

Catalysts like enzymes have proven to be successful molecular recognition agents for continuous sensing [6]. The glucose electrode based on glucose oxidase as the molecular recognition element is but one of several successful examples of this approach. However, we see this approach as limited by the availability of enzymes that recognize different substrates. If you start with an analyte that is not the substrate of an available enzyme, then this approach is not viable.

C. The shotgun approach

What we call the “shotgun” approach is one of two general strategies for preparing molecular recognition elements. The “shotgun” approach involves preparing a large library of potential molecular recognition elements with different conformations. This library is then exposed to analyte. Those members of the library that bind to analyte are then separated from the rest of the library and amplified. A typical library may have as many as 10^12^ to 10^14^ different members. However, only a few of them will have the appropriate conformations to interact strongly with analyte.

This is the strategy employed to develop “aptamers”, i.e., single nucleotide chains that selectively bind analyte. It is also the strategy employed by our natural immune systems. We have ca. 10^12^ different antibodies waiting to interact with foreign invaders. When we do get a foreign invasion, only a small fraction of our antibodies will bind strongly to the invader. These are then amplified so that we have enough antibodies to neutralize the foreign invader. These two approaches are considered separately below:

- Aptamers: Aptamers are single strands of oligonucleotides, DNA or RNA [8,9]. The nucleotide pairs, thymine and adenine (A and T), and, cytosine and guanine (C and G), have structures that allow them to hydrogen bond strongly to each other. A single oligonucleotide strand will adopt a minimum energy conformation that allows the maximum degree of interaction. Different oligonucleotide strands will adopt different conformations that depend on their sequence.

The first step in preparing an aptamer is to make a large library of different DNA sequences. This library typically contains 10^14^ to 10^16^ members. The next step is to pick out the few sequences that selectively bind to the analyte of interest. This is done by what is called the SELEX process. This stands for Selective Enrichment of Ligands by Exponential Enrichment. It is a tedious process that involves multiple affinity chromatography steps with a stationary phase that involves an analyte bound to a solid support. It includes at least one affinity step with just the solid support in order to remove sequences that are binding to the solid support rather than the analyte. However, once a sequence that binds strongly to a particular analyte has been identified, then this can be amplified and incorporated into a sensing element.

Aptamer development is a large topic with many recent articles and many potential applications. To generalize about aptamer properties risks missing important exceptions. Typical K_D_ values can be in the micromolar range for small molecules binding to aptamers and rarely are below 10 nM [10,11].

Because K_D_ values are often larger than required to measure low analyte concentrations, chemical modification of aptamers with the goal of reducing K_D_ values is an active area of research [9,12].

Generalizing about the kinetic properties of aptamer binding is even more difficult than generalizing about the magnitude of affinity constants. These rates will depend both on the number of collisions between aptamer and substrate as well as on the fraction of collisions that have the right orientation for the reaction to occur. As the substrate that binds to an aptamer gets larger, it diffuses more slowly resulting in fewer collisions per unit time. Furthermore, the larger the substrate, the lower the probability that a collision will have right orientation for binding to take place. As a result, the rate constants for binding get smaller as substrates get larger. Nevertheless, rates for substrate binding by aptamer are quite rapid with second order rate constants greater than 10^4^ M^−1^ s^−1^ [10]. Aptamers have been used as the equilibrium binding molecular recognition agents in reversible chemical sensors that respond continuously to substrate concentration [13,14].

b. Antibodies: Antibodies are proteins that are synthesized by the mammalian immune system. Their overall molar mass is ca. 150,000 daltons. They have a Y-shaped constant region with variable regions that are localized in the tips of the arms of the Y. There are on the order of 10^12^ circulating antibodies in blood. Each of these has a different variable region. When a foreign substance is introduced, some of these antibodies have the appropriate shape for binding. These antibodies then multiply, identifying the foreign substance as the first step in a reaction sequence that leads to the destruction of the invader.

Antibody formation is a general approach because antibodies are formed in response to any foreign invader. Only small molecules that are injected into a mammal fail to elicit antibody production. However, this problem is easily overcome by conjugating a small molecule target with a carrier that is large enough to induce an immune response. When subjected to a foreign invader, several different antibodies generally have the appropriate structure for binding. A single antibody may be prepared by fusing an antibody producing cell with a myeloma cell to produce a hydridoma cell that can be cultured to produce monoclonal antibodies. These are identical antibodies that all have the same binding affinity and kinetics.

Dissociation constants for antibodies vary over a wide range from more than 1 nanomolar to close to 1 picomolar [14]. They are orders of magnitude lower than binding constants for aptamers. This reflects the difference between amino acids and nucleotides. The 20 naturally occurring amino acids have a range of side chains that can interact electrostatically, via hydrogen bonding, hydrophobically, or by acid-base attractions. The dissociation constant depends both on the number of interactions between the foreign invader and their strength.

The second order rate constant for antibody binding to antigen is typically on the order of 1 × 10^5^ M^−1^ s^−1^ [14]. The order of magnitude is similar to that of second order rate constants for aptamer binding. This means that most of the difference in aptamer and antibody affinity corresponds to a difference in the rate of the back reaction. Antibodies are the dominant recognition element for on-site analysis but the slow dissociation rate has precluded their use for continuous analysis. The slow dissociation kinetics necessarily require impractically long response times.

Binding by antibodies or by aptamers does not directly lead to a change in properties that can be easily measured. Surface plasmon resonance does measure the change in refractive index that occurs when large molecules bind to surface bound antibodies. Most other methods require some form of labelling. The same is true of aptamers.

## 5. Templating

The other general approach that can be applied with most potential analytes is templating. The molecule to be determined is reacted with monomers that interact with the template. These are known as functional monomers. Other monomers are included to fill out and crosslink the polymer. This approach is known as molecular imprinting [15]. The materials that are produced by this approach are known as molecularly imprinted polymers. They are most commonly known by the acronym MIPs.

When working with smaller molecules, it is most common to carry out templating in nonhydrogen bonding solvents. Hydrogen bonding is a strong noncovalent interaction between a template and an MIP. It is usually desirable for the functional monomer and the template to interact with each other via hydrogen bonding. If the templating were carried out in water, a strong hydrogen bonding solvent, then the template is most likely to hydrogen bond with the solvent rather than with a functional monomer.

When working with larger molecules that are not soluble in nonhydrogen bonding solvents, other approaches have been developed [16,17].

As measured by the number of publications in this area, MIPs have been quite successful. However, we consider this to be misleading. Most analytical applications that use antibodies continue to use antibodies. The reason for the large number of publications is that MIPs are easy to prepare and usually do bind the template with some selectivity. However, they fail with respect to the two figures of merit considered here. Dissociation constants when measured are usually on the order of 10^−6^ M and binding kinetics are usually so slow that binding takes many minutes or even hours to be complete.

The problem seems to be the use of covalent crosslinks in the MIP formulations. These are included to preserve the conformation of the binding side. However, a high degree of covalent crosslinking necessarily leads to a separate highly crosslinked polymer phase. The slow binding kinetics almost certainly arise because the template has to diffuse into the MIP phase to reach available binding sites. This is a slow process, even when the MIP phase is formulated to be very thin so that the template only has to travel a short distance to access all the binding sites.

The high dissociation constant is likely due to the rigidity of the resulting material. This prevents the MIP from accommodating to the shape of the template and maximizing its interactions with the template to increase the Gibbs free energy change associated with binding.

## 6. Templating with Noncovalent Crosslinks

The main purpose of this perspective has been to put our own research into context. We believe that templating can be a useful approach for producing polymers that bind selectively. However, we believe that the widespread use of covalent crosslinks is the reason that MIPs prepared to date have not come close to having the same binding properties as aptamers or antibodies.

Crosslinking seems to be essential for preparing materials that selectively bind the template. In both aptamers and antibodies, most or all of the crosslinks are not covalent. In aptamers the crosslinks are hydrogen bonding interactions between complementary nucleotides, adenine with thymine and cytosine with guanine. In antibodies, several types of crosslinks are possible including hydrophobic interactions between amino acids with hydrophobic side groups, acid-base interactions between an acidic and a basic side group and electrostatic interactions between positively and negatively charged side groups. All of these are noncovalent.

Our approach is to combine the convenience of templating with the enhanced binding affinity and kinetics that are observed with noncovalent crosslinks. While this research is still in its early stages, we have preliminary data confirming that templated polymers prepared with predominantly noncovalent crosslinks bind with affinity and kinetics that improve on aptamers. We also have preliminary data showing that binding induces a conformational change in the polymer, a phenomenon that we can exploit to get a direct readout of the extent of binding.

We have also identified the main problem that we need to solve to make this approach practical. When we make such a polymer as a solid, we are able to dissolve it in water. However, measurements of the extent of binding show relatively little binding. However, when we dilute the polymer, the binding stays the same or increases. This has led us to conclude that our polymer chains are tangled. To make this approach practical we need to find a way to prepare untangled chains. This is currently a major focus of our research.

Our initial system involved fluorescein as the template. This template was chosen because fluorescein can be easily detected at low concentrations by fluorescence. It is not an important analyte to measure in its own right. The polymer chain was mostly poly(*N*-isopropylacrylamide) (pNIPAM). This polymer was chosen because of previous work showing that lightly crosslinked pNIPAM not only can be templated but also undergoes a conformational change when binding the template [18]. It makes sense to us that the hydrophobic isopropyl groups will tend to associate with each other, thus forming noncovalent crosslinks. We have successfully prepared polymers with a pNIPAM backbone and acid-base crosslinks (the formulation includes both methacrylic acid and vinyl pyridine) and electrostatic crosslinks (the formulation includes both a cationic and anionic monomer). Most of our formulations include 2 mole-% covalent crosslinker, *N*,*N*’-methylenebisacrylamide. The rationale behind this choice is that a fixed crosslinker along with at least two other kinds of noncovalent crosslinkers will assure that the polymer always returns to the same conformation, i.e., the conformation with the template binding site. Whether or not this is important for binding and selectivity remains an open question. What we do know is that our polymers dissolve in water rather than forming a separate solid phase as do MIPs that include much higher percentages of covalent crosslinker.

A key aspect of our research is the use of reverse addition fragmentation transfer (RAFT) for polymer synthesis [19]. This allows us to control the length of our polymers by controlling the ratio of polymerizable monomers to the chain transfer agent required for RAFT. RAFT also allows us to introduce end groups onto our polymer, including electroactive compounds for voltametric detection and fluorophores for optical detection. This is accomplished by first synthesizing RAFT chain transfer agents that include these molecules. However, if we modify a RAFT agent we need to do control experiments to confirm that the modified chain transfer agent still has the appropriate reactivity for polymerizing *N*-isopropylacrylamide.

Typically, we work with polymers that have a nominal length of 100. This is short enough to allow us to use NMR to measure polymer length by comparing the area due to backbone monomer resonances to the area for a resonance arising from the charge transfer agent. However, this is only possible when we use charge transfer agents that have resonances that do not overlap polymer resonances.

The solvent we have been using is dioxane. Dioxane freezes at a relatively high temperature, simplifying the use of freeze-pump-thaw sequences to remove dissolved oxygen prior to polymerization. Once the polymer is formed, it can be precipitated out using hexane. The resulting polymer can be collected by vacuum filtration and dried.

We have bound our fluorescein templated polymer to gold nanoparticles. Using the fluorescein templated polymer attached to a gold surface, we have measured the dissociation constant at ca. 3 nM, a value that not only improves upon other MIPS but also most aptamers. Because binding to the gold bound polymer quenches fluorescence, we were also able to show that binding was complete in less than 2 s, much faster than typical MIPs.

In collaboration with Prof. Ed Song and his research group in the Dept. of Electrical and Computer Engineering at the University of New Hampshire, we have also performed preliminary experiments with 4-nitrophenol templated polymer on the surface of a gold electrode [20]. Polymers prepared by RAFT can be reduced to thiols that covalently bond to a gold surface. When this was performed on our polymer, it took about 30 days for the electrical properties of the gold surface to stabilize. We attribute this to untangling of polymers chains that are covalently bound to the gold surface from chains that are not bound to the surface. Once this surfaced stabilized, experiments showed that nitrophenol binding by the surface bound templated polymer affected ferricyanide access to the electrode surface. This observation suggests that template binding causes a conformational change.

That we observe a conformational change upon template binding suggests that we can use this phenomenon to measure the extent of binding. One approach that we are actively exploring is to include both a donor and an acceptor fluorophore in the polymer chain and to measure the extent of fluorescence resonance energy transfer (FRET) as a function of templating binding. The assumption is that the conformational change will modify the distance between donor and acceptor and thus affect the extent of FRET.

## 7. Conclusions

This purpose of this perspective has been to put our research in context. Our goals are to achieve high affinity, rapid binding using noncovalently crosslinked polymers. Preliminary results using fluorescein as a template confirm binding with high affinity. The value of K_D_ for fluorescein binding is 3 nM, higher than most template binding constants. We believe that noncovalent crosslinks lead to binding sites that are better able to organize around the template.

Our preliminary results also confirm that much faster binding kinetics when templated polymers are prepared using noncovalent crosslinks. We attribute this result to the increased flexibility of noncovalent crosslinks. They can easily change conformation to accept template into the binding cavity.

While our preliminary experiments suggest that we can achieve our goal of high affinity rapid binding templated polymers that are suitable for molecular recognition in sensors, the practical utility of these materials will be much greater if we can find a convenient, fast method for preparing polymer chains that are not tangled.

## Data Availability

Not applicable.
